# Endurance training or beta-blockade can partially block the energy metabolism remodeling taking place in experimental chronic left ventricle volume overload

**DOI:** 10.1186/1471-2261-14-190

**Published:** 2014-12-17

**Authors:** Dominic Lachance, Wahiba Dhahri, Marie-Claude Drolet, Élise Roussel, Suzanne Gascon, Otman Sarrhini, Jacques A Rousseau, Roger Lecomte, Marie Arsenault, Jacques Couet

**Affiliations:** Groupe de recherche sur les valvulopathies, Centre de Recherche, Institut Universitaire de cardiologie et de pneumologie de Québec, Université Laval, 2725, Chemin Sainte-Foy, Québec City, Québec G1V 4G5 Canada; Centre d’imagerie moléculaire de Sherbrooke, Centre de recherché Étienne-LeBel, Centre Hospitalier Universitaire de Sherbrooke, Université de Sherbrooke, Sherbrooke, Canada

## Abstract

**Background:**

Patients with chronic aortic valve regurgitation (AR) causing left ventricular (LV) volume overload can remain asymptomatic for many years despite having a severely dilated heart. The sudden development of heart failure is not well understood but alterations of myocardial energy metabolism may be contributive. We studied the evolution of LV energy metabolism in experimental AR.

**Methods:**

LV glucose utilization was evaluated *in vivo* by positron emission tomography (microPET) scanning of 6-month AR rats. Sham-operated or AR rats (n = 10-30 animals/group) were evaluated 3, 6 or 9 months post-surgery. We also tested treatment intervention in order to evaluate their impact on metabolism. AR rats (20 animals) were trained on a treadmill 5 times a week for 9 months and another group of rats received a beta-blockade treatment (carvedilol) for 6 months.

**Results:**

MicroPET revealed an abnormal increase in glucose consumption in the LV free wall of AR rats at 6 months. On the other hand, fatty acid beta-oxidation was significantly reduced compared to sham control rats 6 months post AR induction. A significant decrease in citrate synthase and complex 1 activity suggested that mitochondrial oxidative phosphorylation was also affected maybe as soon as 3 months post-AR.

Moderate intensity endurance training starting 2 weeks post-AR was able to partially normalize the activity of various myocardial enzymes implicated in energy metabolism. The same was true for the AR rats treated with carvedilol (30 mg/kg/d). Responses to these interventions were different at the level of gene expression. We measured mRNA levels of a number of genes implicated in the transport of energy substrates and we observed that training did not reverse the general down-regulation of these genes in AR rats whereas carvedilol normalized the expression of most of them.

**Conclusion:**

This study shows that myocardial energy metabolism remodeling taking place in the dilated left ventricle submitted to severe volume overload from AR can be partially avoided by exercise or beta-blockade in rats.

**Electronic supplementary material:**

The online version of this article (doi:10.1186/1471-2261-14-190) contains supplementary material, which is available to authorized users.

## Background

The role of impaired myocardial energetics in the development and progression of heart failure (HF) seems to be central [1]. The energy-depletion theory of HF is not new and a multitude of recent studies have provided solid evidence that myocardial metabolism is strongly affected in humans as well as in many animal models of left ventricular hypertrophy and HF [1–3]. Both systolic and diastolic functions seem to be intimately affected by impaired myocardial energetics [4–8].

Alterations of myocardial metabolism caused by chronic valve disease such as aortic regurgitation (AR) are unclear and have not been studied like the ones caused by pressure overload or ischemia [9–19]. Chronic AR is usually well tolerated for many years before HF occurs. AR patients develop severely dilated and hypertrophied hearts but remain in a clinical pre-HF state with a normal LV ejection fraction for long periods of time [20]. The reason why they suddenly progress towards symptoms and HF after this long stable period is not well understood. There is currently no treatment proven effective to decrease AR related morbidity-mortality or delay the evolution towards HF in humans [21]. The only solution available for now remains valve replacement surgery when the left ventricle becomes too dilated, systolic indices progressively decrease or when symptoms occur. Over the years, we have showed that treatment targeting the renin-angiotensin-aldosterone or the adrenergic systems can help reduce LH hypertrophy, maintain cardiac function and improve survival in a rat model of chronic AR [22–25]. We did observe a similar effect by non-pharmaceutical strategy i.e. moderate endurance training [26].

We suggested that AR left ventricles with severe eccentric hypertrophy suffer from significant myocardial metabolic impairment even before systolic dysfunction becomes apparent and observed that as early as 8 weeks post-AR myocardial energy substrate preference was altered and a switch toward increased glucose utilization was observed [27].

Here, we studied the long-term alteration in LV energy metabolism associated with chronic volume overload caused by severe AR in Wistar rats. We show that treatments (training and beta-blockade) that reduce LV dilatation and help maintain function are also associated with a normalization of the energy metabolism.

## Methods

### Animals

Six groups of Wistar male rats (350–375 g) were studied for either 90, 180 or 270 days. For each end-point time, the animals were divided in two groups: sham-operated animals (sham) or surgically induced AR. All groups consisted of 15 animals with the exception of the 270-day AR group consisting of 30 animals. An additional group (n = 10) of young healthy rats served as controls. For the μPET study, eight additional animals (4 shams and 4 AR) were studied 6 months after surgery. For endurance training protocol, a group of 20 animals were exercised 5 days/week for 270 days on a motorized treadmill with a slope of 10°. The duration and the intensity increased progressively during the first 8 weeks until the animals were running for 30 minutes at 20 m/min as previously described [26]. The influence of beta-blockade was tested using carvedilol in four groups of male Wistar rats (15 animals/group): sham and AR animals receiving or not carvedilol (30 mg/kg/d in drinking water). Training or carvedilol were started two weeks post-surgery for six months. The protocol was approved by the Université Laval’s Animal Protection Committee and followed the recommendations of the Canadian Council on Laboratory Animal Care. The animal PET imaging protocol was approved by the Animal Ethics Committee of the Faculty of Medicine of the Université de Sherbrooke. Severe AR was induced by retrograde puncture of the aortic valve leaflets as previously described [28]. At the end of the protocols, surviving animals were sacrificed, hearts were quickly dissected and all cardiac chambers were weighed. LV was snap-frozen in liquid nitrogen and kept at -80°C for further analysis. All sacrifices were scheduled at similar times of the day to avoid circadian variations.

### Echocardiography

A complete M-Mode, 2D and Doppler echocardiogram was performed on the animals under 1.5% inhaled isoflurane anesthesia using a 12 MHz probe with a Sonos 5500 echograph (Philips Medical Imaging, Andover, MA) immediately before and during surgery, after 2 weeks, 3, 6 and 9 months as previously described [26].

### Small animal PET protocol

Imaging experiments and data analysis were performed essentially as described before [29–32] on a LabPET™ avalanche photodiode-based small animal PET scanner (Gamma Medica, Northridge, CA) at the Sherbrooke Molecular Imaging Centre. [^18^F]-fluorodeoxyglucose ([^18^F]-FDG) (30–40 MBq, in 0.3 ml plus 0.1 ml flush of 0.9% NaCl) was injected via the caudal vein over 30 s. A 45-min dynamic PET data acquisition followed by a 15-min static acquisition was done to determine glucose utilization [myocardial metabolic rate of glucose (MMRG)] using multicompartmental analysis as previously described [32, 33]. The static scan served to draw regions-of-interest (ROIs) on each segment of the LV wall. Blood samples were taken before and after the scans to determine an average blood glucose level.

### Analysis of mRNA accumulation by quantitative RT-PCR

The analysis of LV mRNA levels by quantitative RT-PCR has been described in details elsewhere [26].

### Enzyme activity determination

Enzyme activity assays are described in details in the supplementary section (Additional file 1, Methods) [25, 27, 34].

### Statistical analysis

Results are presented as mean ± SEM unless specified otherwise. Inter-group comparisons were done using Student’s t-test or Mann–Whitney t-test for PET protocol. One-way or two-way ANOVA were also used for the analysis of data when required. Statistical significance was set at a *p* < 0.05. Data and statistical analysis were performed using Graph Pad Prism version 6.04 for Windows, Graph Pad Software (San Diego, CA).

## Results

**Figure 1:**
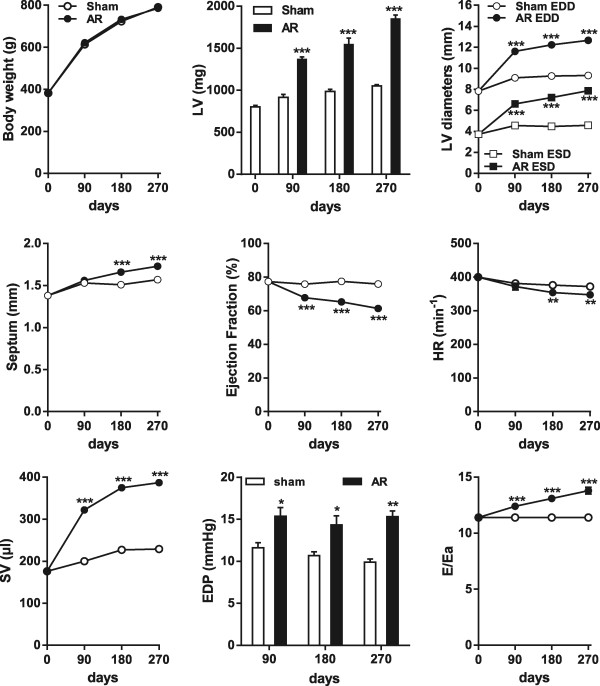
**Evolution of LV remodeling as evaluated by echocardiography in experimental volume overload from severe aortic valve regurgitation in Wistar rats.** LV dimensions, ejection fraction (EF), heart rate (HR), stroke volume (SV) and ratio of early transmitral velocity to tissue Doppler mitral annular early diastolic velocity (E/Ea ratio) were evaluated throughout the course of the protocol as assessed by echocardiography in sham-operated animals (sham: white circles or bars) and AR rats (AR: black circles or bars) at the beginning of the protocol, after 90, 180 and 270 days. Body weight was also recorded at the time of echocardiography. Left ventricular wet tissue weight was evaluated at sacrifice. End-diastolic pressures (EDP) were evaluated by direct LV catheterization prior euthanasia. LV weight, EDD: end-diastolic diameter, ESD: end-systolic diameter, Septum: septal wall thickness. Results are reported in as mean ± SEM (n = 10–15 per group). *p < 0.05, **p < 0.01 and ***p < 0.001 between sham and AR groups.

All sham-operated animals were alive at the end of the protocol. After 3, 6 and 9 months, 14/15, 12/15 and 14/30 animals were still alive in the AR groups, respectively. As illustrated in Figure 1, no differences in body weight were observed between the sham and AR groups. Overall growth was similar between groups (similar tibial lengths, results not shown). LV wet tissue weights were significantly increased in the AR groups compared to controls and this increase was steady over the 9 month period.

### Echocardiographic data

The echocardiographic data from all study groups are also presented in Figure 1. End-diastolic (EDD) and end-systolic diameters (ESD) sharply increase during the first 3 months and continue to increase but at a slower pace thereafter. AR animals have a lower ejection fraction than normal sham animals. Ejection fraction slowly decreases over the 9 months but it still remains within what is considered a normal range (above 60%). The end result after 9 months of chronic severe AR is a severely dilated ventricle with eccentric hypertrophy and relatively preserved ejection fraction. AR animals have as expected an increased stroke volume compared to normal sham animals whereas their heart rate is slightly diminished. Diastolic echocardiographic parameters were also measured. AR animals had a significantly higher E/Ea ratio than sham animals suggesting increased left ventricular end-diastolic pressures. This correlated well with the invasive LV end-diastolic pressures (EDP) measurements that were also increased in the AR groups.

### Markers of hypertrophy and extracellular matrix remodeling

**Figure 2:**
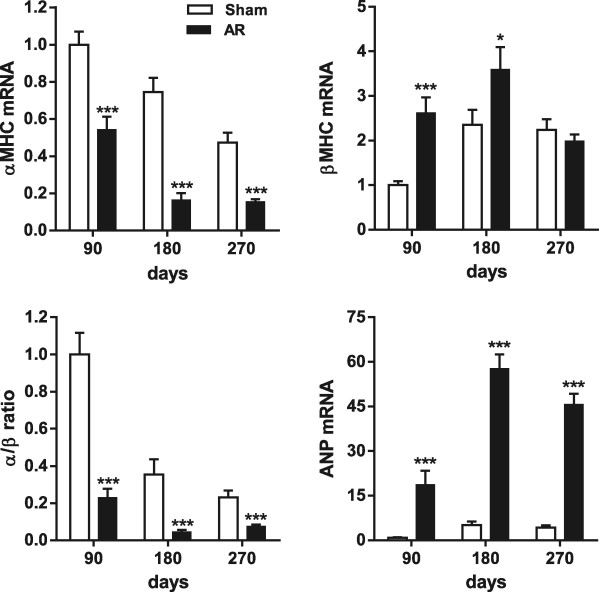
**Evaluation by real-time quantitative RT-PCR of the LV mRNA levels of genes related to LV hypertrophy.** *p < 0.05 and ***p < 0.001 between sham and AR groups. Sham (sham-operated animals) at 90 days post-surgery group mRNA levels were normalized to 1. ANP, atrial natriuretic peptide; αMHC, myosin heavy chain alpha; βMHC, myosin heavy chain beta; α/β: ratio of the two MCH forms.

The relative gene expression of both the alpha and beta forms of myosin heavy chains was modified in AR animals in which the alpha/beta ratio was strongly reduced (Figure 2). As expected, ANP gene expression was elevated in AR animals.

### Myocardial glucose consumption

**Figure 3:**
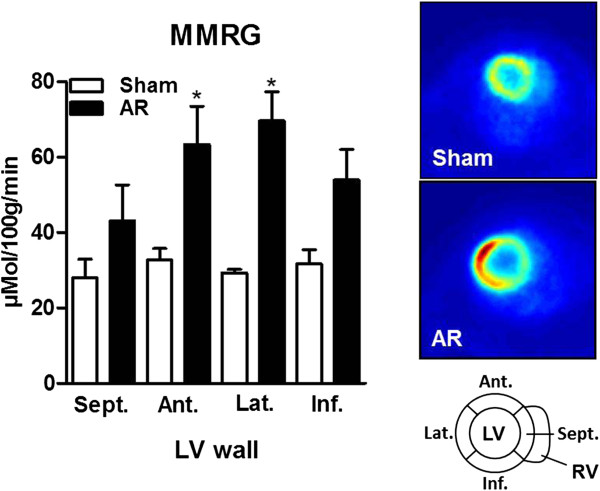
**In vivo glucose uptake by the left ventricle of AR and sham rats as evaluated by micro positron emission tomography (μPET).** Myocardial metabolic rate of glucose (MMRG) was evaluated as described in the Material and Methods section for each segment of the LV wall as schematized in the bottom right of the figure. Regional MMRG evaluation was realized in four different animals per group and results were expressed as the mean ± SEM. *p < 0.05 between sham and AR groups. Sept: septal wall, Ant: anterior wall, Lat: lateral wall and Inf: inferior wall. At the right of the column graph, representative transaxial μPET scan images after injection of [^18^F]-FDG are illustrated.

Micro-PET imaging was used to investigate how glucose consumption was altered *in vivo* in AR rats after 6 months of severe volume overload. Regional myocardial metabolic rate of glucose (MMRG) was estimated from the dynamic uptake of [^18^F]-FDG after intravenous bolus injection using μPET. As illustrated in Figure 3, MMRG was increased in AR myocardium and this increase was preferentially located to the LV free wall (anterior and lateral).

### Myocardial metabolic enzymes

**Figure 4:**
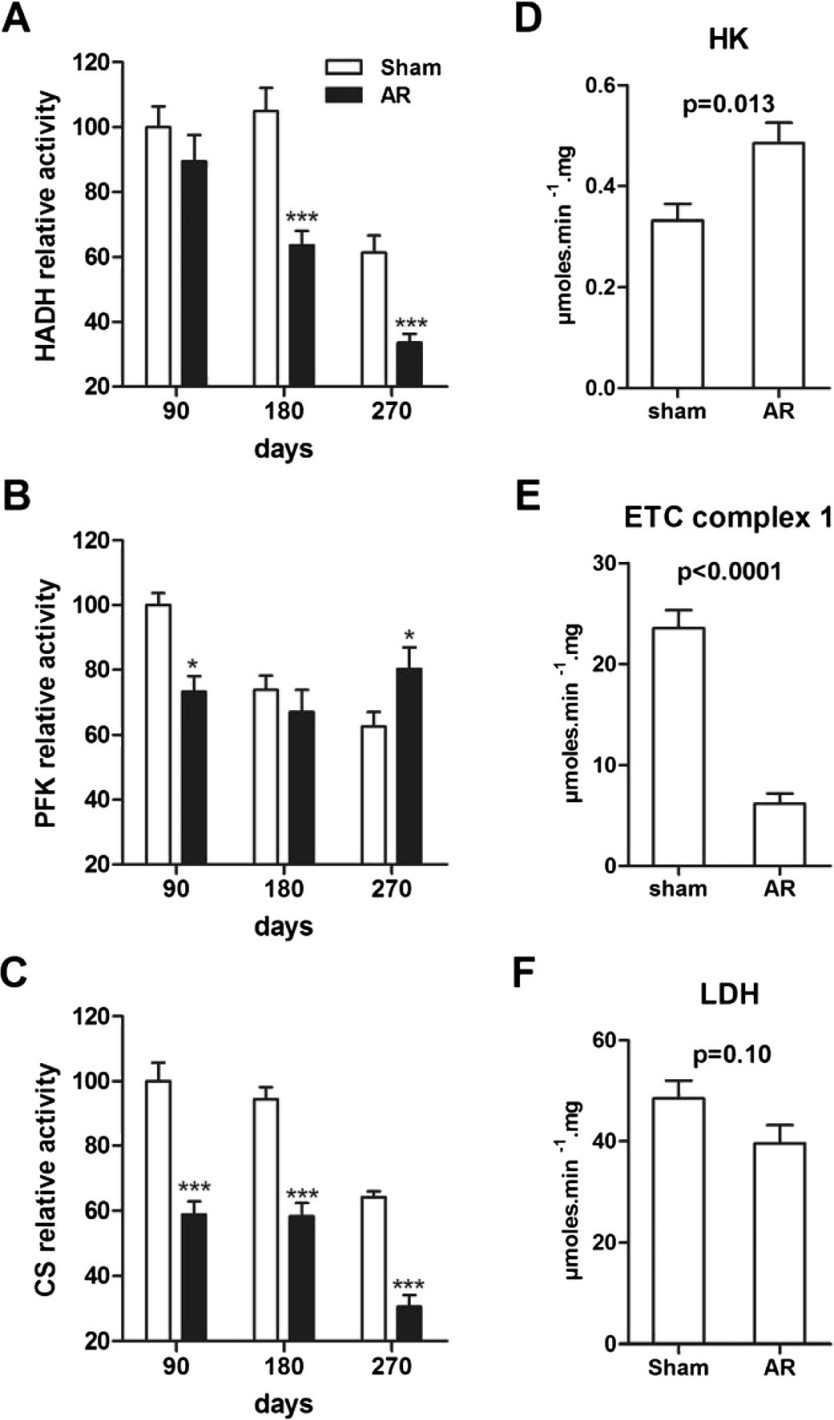
**LV myocardial activity levels of enzymes implicated in fatty acid β-oxidation, glucose metabolism and mitochondrial energy production in 9-month AR rats and relative evolution over time.** HADH (hydroxyacyl-Coenzyme A dehydrogenase; **A)**, PFK (phosphofructokinase; **B)**, citrate synthase (CS; **C**) enzymatic activities were measured in LV homogenates from at least 10 animals in each group as described in the Methods. Hexokinase (HK; **D**), complex 1 (ETC. complex 1, rotenone-sensitive activity; **E)** and LDH (lactate dehydrogenase **(F)** activities were measured in LV homogenates from 10 270-day animals. Results are reported relative to activity level measured in 90-day sham rats **(A, B and C)** or in μmoles/min/mg of tissue **(D, E, and F)** or. *p < 0.05, **p < 0.01 and ***p < 0.001 between sham and AR groups.

We measured enzymatic activity levels in LV crude homogenates (Figure 4). The HADH (hydroxyacyl-Coenzyme A dehydrogenase) responsible for fatty acid β-oxidation was less active in the AR group after 9 months compared to sham animals. Normal aging also reduced HADH activity levels in the shams after 9 months but much less than in chronic AR. Normal aging was accompanied by a steady decrease in the activity level of the glycolytic enzyme phosphofructokinase (PFK) whereas it remained stable in AR animals over the 9 month follow-up. This resulted in a higher PFK activity level in AR animals after 9 months compared to age-matched sham animals. The entry of acetyl-CoA in the citric acid cycle is catalyzed in the mitochondria by the citrate synthase (CS). Again, normal aging was accompanied by a decrease in CS activity levels. CS activity levels were however significantly lower in AR animals after 3, 6 and 9 months when compared to aged-matched sham animals. The first step of glycolysis is catalyzed by the hexokinase (HK). HK activity levels were significantly increased in all AR animals compared to the shams after 9 months. On the other hand, the first step in the electron transfer chain (mitochondrial ETC. complex 1) was strongly reduced in AR rats compared to sham after 9 months while lactate dehydrogenase levels were not significantly changed.

### Endurance training can help normalize myocardial metabolic enzymes

**Figure 5:**
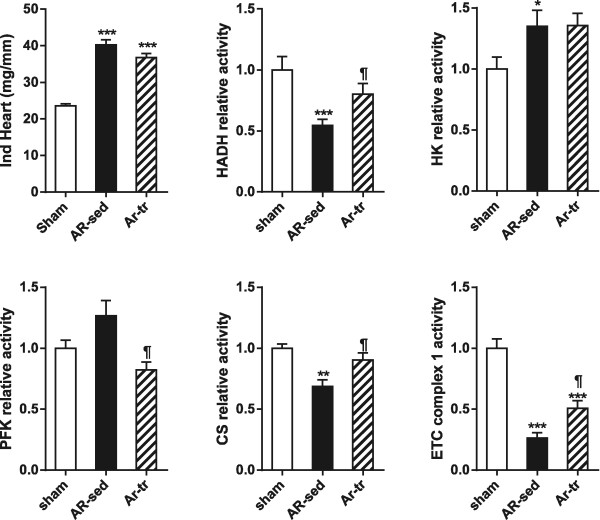
**Moderate endurance training (tr) helps normalize activity levels of enzymes implicated in the LV energy metabolism in 9-month AR rats.** Indexed (i) heart weight was corrected for the tibial length. HADH (hydroxyacyl-Coenzyme A dehydrogenase), HK (hexokinase) PFK (phosphofructokinase), citrate synthase (CS) and complex 1 enzymatic activities were measured in LV homogenates as described in the Methods. Results are expressed as mean ± SEM (n = 10/group) in μmoles/min/mg of tissue. *p < 0.05, **p < 0.01 and ***p < 0.001 between sham and AR and ¶p < 0.05 between AR and AR-tr groups.

In order to evaluate if some alterations of the myocardial energy metabolism could be reversed, we tested the impact of moderate endurance training we previously showed to improve the condition of chronic AR rats. AR rats were thus submitted to moderate intensity endurance training on a treadmill (up to 20 m/s for 30 minutes) for a period of 9 months. Of the 20 animals, 14 survived the entire protocol. As illustrated in Figure 5, endurance training did not reduce the heart hypertrophy in AR animals although a trend was observed. Levels of enzymatic activity were normalized for the HADH, CPT, PFK and CS suggesting an improvement of the myocardial metabolic profile associated with exercise.

### Endurance training does not reverse the down-regulation of genes associated with energy metabolism in AR

**Figure 6:**
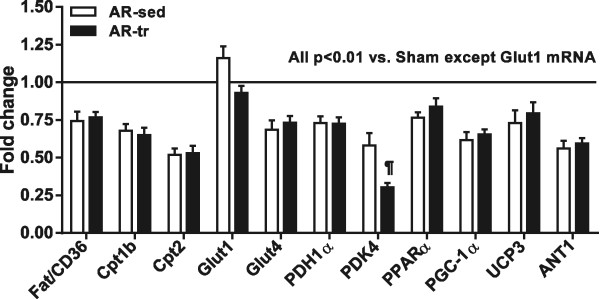
**Evaluation by real-time quantitative RT-PCR of the LV mRNA levels of 11 genes related to cardiac metabolism in 9-month rats and impact of endurance training.** Results are reported in arbitrary units as mean ± SEM (n = 15/gr). Levels in sham animals were fixed to 1. FAT/CD36: fatty acid transporter/CD antigen 36, CPT1b: carnitine palmitoyltransferase 1b and CPT2: carnitine palmitoyltransferase 2, Glut1: glucose transporter 1, Glut4: glucose transporter 4, PDH1a: pyruvate dehydrogenase 1 alpha and PDK4: pyruvate dehydrogenase kinase 4, PPARα: peroxisome proliferator activator receptor alpha, PGC-1α: Peroxisome proliferator-activated receptor gamma coactivator-1-alpha, UCP3: uncoupling protein 3 and ANT: adenine nucleotide translocase. P values are indicated above each bar compared to sham controls. ¶p < 0.05 between AR and AR-tr groups.

The results of the 9 month AR gene expression levels of various enzymes and transporters related to fatty acid and glucose metabolism in the myocardium compared to age-matched sham animals as well as the effects of training are summarized in Figure 6. FAT/CD36 gene expression (responsible for fatty acids transport into the cell) as well as those of CPT1b and CPT2 (responsible for the entry of fatty acids in the mitochondrion), were all decreased in AR animals. Glucose transporters (GLUT) 1 and 4 mediate glucose entry in the cell. GLUT4 mRNA expression levels were decreased by about 25% in AR animals whereas mRNA levels encoding for GLUT1 remained unchanged. The formation of acetyl-CoA from pyruvate is catalyzed by the pyruvate dehydrogenase complex. We evaluated the gene expression of one member of this complex (PDH1α) as well as one of its inhibitors (PDH kinase 4 or PDK4). The expression of those two genes was significantly down-regulated in AR animals. One main regulator of fatty acid oxidation is the peroxisome proliferator-activated receptor alpha (PPARα). PPARα mRNA levels were lower in AR animals after 9 months. The mechanism by which PPARα activates a mitochondrial biogenic response involves one of its inducible co-activator: the peroxisome proliferator-activated receptor gamma coactivator-1-alpha or PGC-1α. The mRNA levels encoding for this gene was also markedly reduced in our AR animals. The same was true for the gene expression of the uncoupling protein 3 (UCP3). We also evaluated ANT1 (adenine nucleotide translocase 1) which is known to facilitate the exchange of extra-mitochondrial ADP with mitochondrial ATP. We observed again a strong decrease in the expression of this gene in the AR animals compared to the sham controls. Training did not modulate gene expression in AR rats for the molecules evaluated.

**Figure 7:**
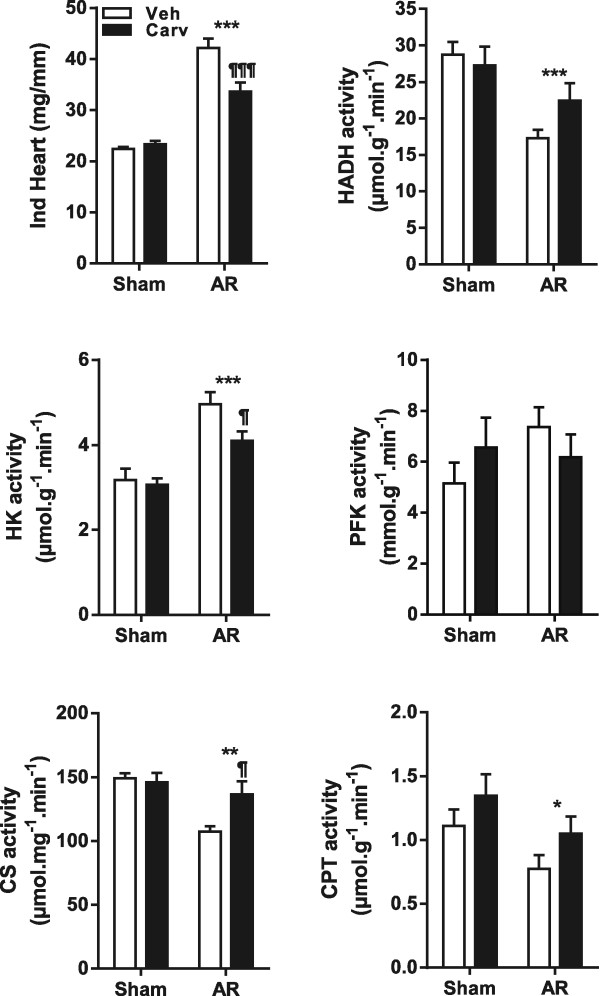
**Beta-blocker carvedilol treatment helps normalize activity levels of enzymes implicated in the LV energy metabolism in 6-month AR rats.** Indexed (i) heart weight was corrected for the tibial length. HADH (hydroxyacyl-Coenzyme A dehydrogenase), HK (hexokinase) PFK (phosphofructokinase), citrate synthase (CS) and carnitine palmitoyl transferase (CPT) enzymatic activities were measured in LV homogenates as described in the Methods. Results are expressed as mean ± SEM (n = 9-12/group) in μmoles/min/mg of tissue. *p < 0.05, **p < 0.01 and ***p < 0.001 between sham and AR groups and ¶p < 0.05 between AR and AR-Carv groups.

**Figure 8:**
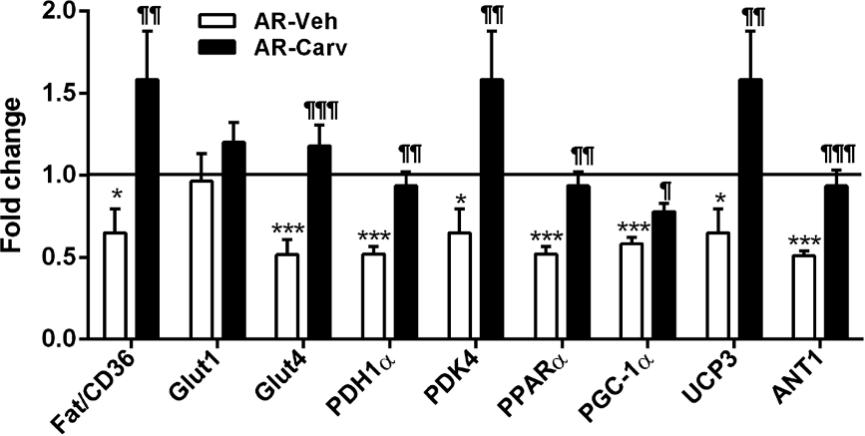
**Carvedilol reverses down-regulation of genes implicated in cardiac energy metabolism in 6-month AR rats.** Results are reported in arbitrary units as mean ± SEM (n = 15/gr). Levels in sham animals were fixed to 1. FAT/CD36: fatty acid transporter/CD antigen 36, Glut1: glucose transporter 1, Glut4: glucose transporter 4, PDH1a: pyruvate dehydrogenase 1 alpha and PDK4: pyruvate dehydrogenase kinase 4, PPARα: peroxisome proliferator activator receptor alpha, PGC-1α: Peroxisome proliferator-activated receptor gamma coactivator-1-alpha, UCP3: uncoupling protein 3 and ANT: adenine nucleotide translocase. P values are indicated above each bar compared to sham controls. ¶p < 0.05 between AR and AR-tr groups.

A six-month carvedilol treatment improves the energy metabolism enzyme activity levels as well as the expression profile of metabolic genes in AR rats (Figures 7 and 8).

At the end of the 6-month protocol, all sham-operated treated or not with carvedilol were alive while 9/15 and 12/15 rats were still present in the AR-Veh and AR-Carv groups, respectively. LV hypertrophy was present in both AR groups but significantly less in the animals treated with carvedilol (Figure 7). This was also true for the size of cardiac myocytes as evaluated in LV sections (Additional file 1: Figure S1).

As illustrated in Figure 7, the carvedilol treatment partially reversed the changes in HADH, hexokinase, citrate synthase and complex 1 associated with eccentric LHV in AR rats.

The same was true for the LV mRNA levels of a number of genes associated with energy metabolism where the general down-regulation was mostly reversed by carvedilol.

## Discussion

Factors influencing the development and evolution of LV remodelling in AR are poorly understood. Here, we provide a longitudinal study focusing on myocardial energy metabolism in the LV of rats with chronic severe AR.

The heart is in a constant need of energy substrates since it does not maintain significant reserve [1]. The myocardial energetic machinery is complex and can be affected at many interacting levels including: substrate utilization/preference, oxidative energy production in the mitochondria, energy transport and consumption by the contractile myofibrils [35].

Our experimental model causes severe LV dilatation. Despite the presence of important hypertrophy in this rat model, HF remains a late occurring event as seen in humans [36]. As we have previously reported, the majority of AR rats have a systolic function within normal range (ejection fraction > 60%, normal dP/dt+) even after 9 months [26]. Diastolic abnormalities become clearly evident as soon as 2 or 3 months after AR induction [34, 37].

As reported by others in models of LV concentric hypertrophy and of HF, we too observed a shift in the ratio of the gene expression of myosin heavy chains α and β in AR rats. This also occurs but much less, with normal aging [38]. This shift can significantly affect the energetic efficiency of the heart and could point towards an imminent shift towards HF.

In this study, we showed in vivo using μPET that the LV myocardium of AR rats increased its glucose consumption. This increase seems to be more pronounced in the LV free wall mostly in the lateral and anterior portions. It could be suggested that dilation may not be homogeneous through the LV was and that the observed metabolic changes in the LV myocardium may reflect this. We already observed the opposite situation in the AR rat where fatty acid uptake was reduced in the same LV region where we now observe an increase in glucose uptake [25]. Concentric LV hypertrophy is associated with a shift in substrate preference from free fatty acids to glucose [35]. Our μPET results confirm this for our animals in vivo with eccentric VO LVH.

We also described the impact of normal aging on the levels of enzymatic activity related to myocardial metabolism. We detected a significant loss of myocardial activity for three central metabolic enzymes (HADH, CS and PFK) due to normal aging. The activity levels of these enzymes decreased by at least 25% in the last six months of the protocol. It is possible though that these changes reflect a progression from a stage of global body growth at a younger age to the more stable adult stage. Adding AR amplified this effect on HADH and CS activities. This suggests that fatty acid oxidation is further impaired in the late stage of AR and that the total mitochondrial oxidative capacity of the myocardium may then be less than normal [10]. On the other hand, PFK activity remained stable in the hearts of AR animals suggesting a shift towards glucose utilization as previously seen in concentric LV hypertrophy and HF [39]. μPET imaging also confirmed this hypothesis. Our data show a decrease in fatty acid transport-related Fat/CD36 in the animals with AR. Carnitine palmitoyl-transferase gene expression and enzymatic activity was also decreased. These observations are consistent with data published in other models of LVH [11, 40]. The mitochondrial energetic machinery also seems to be affected by the LV volume overload as shown not only by the decrease in CS activity but also by the strong decrease in the activity of the ETC. complex I in 9-month AR animals. These mitochondrial enzymatic abnormalities could result in myocardial energy starving either in the basal state or in response to an acute stress such as exercise or ischemia. It has been previously reported that VO could induce an inappropriate response to various stresses in two different animal models during the compensated phase of the disease [16, 19]. The down-regulation of ANT1 is another clue pointing towards an abnormal exportation of ATP from the mitochondrion [41] which seems to be seriously impaired in our AR animals after 9 months. The gene expression of PDH1α which is responsible for pyruvate entry into the mitochondria was reduced in AR animals after 9 months compared to normal age-matched controls. Myocardial energetic status at this late stage of the disease in our AR animals probably shares similarities to the one seen in established HF even if systolic function remains in the normal range in our animals.

This study also clearly shows that regular exercise has beneficial effects on the myocardial energetic machinery in this animal model of volume overload cardiomyopathy even before systolic heart failure occurs. These effects were detectable on enzymes and pathways related to fatty acid oxidation and glycolytic capacity as well as to mitochondrial efficiency. The benefits of exercise on LV remodeling, diastolic function and survival we have recently reported could therefore be in part related to improvement in myocardial energetics [26]. One possible mechanism may be via the activation of the IGF1/PI3K/Akt pathway by exercise which can activate survival pathways in cardiac myocytes [42, 43].

We also observed an improvement of myocardial energetics in AR animals treated with the beta-blocker, carvedilol. We had reported that beta-blockade using either metoprolol or carvedilol can reduce the extent of LV hypertrophy development in the rat AR model [23, 36]. The benefits in maintaining systolic function were similar to those observed in endurance-trained animals [26]. It is interesting to observe that although the effects of beta-blockade and exercise were similar at normalizing metabolic enzyme activities, carvedilol treatment also restored gene expression of a number of proteins implicated in the control of substrate uptake and metabolism. This suggests that similarities and differences exist between the mechanisms of action of exercise and beta-blockade. Another possibility is that by a better control of LVH development by carvedilol, many parameters may remain in the normal range.

### Limitations

In this study, we used a range of techniques to evaluate myocardial metabolism in AR rats to demonstrate that substrate preference as well as general energy metabolism is modified in this model and that endurance training and beta-blockade can partially reverse these changes. Obviously, our study can only offer an incomplete portrait of the complex metabolic changes taken place in the myocardium submitted to severe and chronic volume overload. Enzyme activity determinations and gene expression studies made here cannot encompass the wide array of modification in energetics in the hypertrophied myocardium. More thorough studies using μPET in vivo, isolated heart or mitochondria studies could offer supplementary information to better describe these changes.

## Conclusion

Our results clearly show that the myocardium with chronic VO suffers from a significant metabolic stress and develops over time important metabolic abnormalities.

These findings provide for the first time new longitudinal data which may improve our view of the dilated hearts of patients with severe AR. Clinicians currently feel comfortable to follow those patients without any intervention for many years, simply waiting for the LV to become too dilated, for the occurrence of symptoms or until systolic function begins to fall. Based on our findings, we suggest that those volume overloaded hearts develop severe metabolic abnormalities even when systolic function seems preserved. Focusing on myocardial metabolism by various interventions such as targeted drugs, specific diets or exercise may help this metabolically stressed myocardium to improve energy production and maybe prolong the pre-heart failure state significantly. Further studies will be needed to confirm this hypothesis.

## Electronic supplementary material

Additional file 1: **Supplemental methods and data.** This file contains more detailed methods for the enzymatic assays as well as references. In addition, **Figure S1.** is a complement of data for the carvedilol study of Figures 7 and 8 in the manuscript. (PDF 227 KB)
