# Supplementary material for: Endurance training or beta-blockade can partially block the energy metabolism remodeling taking place in experimental chronic left ventricle volume overload
Source: BMC Cardiovasc Disord. 2014 Dec 17;14:190. doi: 10.1186/1471-2261-14-190 (PMC4279960; doi:10.1186/1471-2261-14-190)
Supplement: Supplementary file 1 — Additional file 1: Supplemental methods and data. This file contains more detailed methods for the enzymatic assays as well as references. In addition, Figure S1. is a complement of data for the carvedilol study of Figures 7 and 8 in the manuscript. (PDF 227 KB) [file 12872_2014_832_MOESM1_ESM.pdf]

Endurance training or beta-blockade can partially block the energy metabolism remodeling taking place in experimental chronic left ventricle volume overload.

Supplemental methods and data.

Dominic Lachance[1], Wahiba Dhahri[1], Marie-Claude Drolet[1], Élise Roussel[1], Suzanne Gascon[2], Otman Sarrhini[2], Jacques A. Rousseau[2], Roger Lecomte[2], Marie Arsenault[1] and Jacques Couet[1,3].

1: Groupe de recherche sur les valvulopathies, Centre de Recherche, Institut universitaire de cardiologie et de pneumologie de Québec, Université Laval, Quebec City, Canada.

2: Centre d'imagerie moléculaire de Sherbrooke, Centre de recherché Étienne-LeBel, Centre Hospitalier Universitaire de Sherbrooke, Université de Sherbrooke, Sherbrooke, Canada.

3: Corresponding author: Jacques Couet PhD

Groupe de Recherche en Valvulopathies, Centre de Recherche,

Institut universitaire de cardiologie et de pneumologie de Québec

2725, Chemin Sainte-Foy, Québec, (Québec), Canada, G1V 4G5

Email: jacques.couet@med.ulaval.ca

Methods

Enzyme activity determination: Left ventricle samples were kept at -80°C until assayed for maximal (V_{max}) enzyme activities. Small pieces of LV (20-30 mg) were homogenized in a glass-glass homogenizer with 39 volumes of ice-cold extracting medium pH 7.4 (250 mM sucrose, 10 mM Tris-HCl, 1 mM EGTA). Key enzymes were chosen to represent every major energy-yielding pathway. Hexokinase and phosphofructokinase were chosen for glycolysis, citrate synthase for the citric acid cycle, complex I for the respiratory chain and HADH (hydroxyacyl-Coenzyme A dehydrogenase) and CPT (carnitine palmytoyl transferase) for fatty acid β-oxidation. In addition, electron transfer chain (ETC) complex 1 and lactate dehydrogenase (LDH) were evaluated in selected groups.

HADH (hydroxyacyl-Coenzyme A dehydrogenase), HK(hexokinase) PFK (phosphofructokinase), lactate dehydrogenase (LDH) and CS (citrate synthase) enzyme activities were estimated by the reduction of NADP to NADPH in a spectrophotometer with wavelength set to 340 nm as described in Maltais et al and references within (1, 2).

Activity of the complex I (NADH-ubiquinone oxidoreductase) was evaluated as described by Jarreta et al. (3) with some modifications. Small pieces of LV (20-30 mg) were homogenized in a glass-glass homogenizer with 39 volumes of ice-cold extracting medium. After centrifugation at 15000 x g, supernatant was used for enzymatic assay. The activity was determined in the following reaction medium (1.1mL) (500mM potassium phosphate pH7.5, 50mg/ml BSA, 25mM decyubiquinone); 22.5µl of LV homogenate were added to reaction medium followed by a 5-min incubation at 37°C. The reaction was then initiated by adding NADH to a final concentration of 50µM. Enzyme activity was estimated by the reduction of NADH to NAD^+ in a spectrophotometer with wavelength set at 340nm. To measure the specific complex I activity, the same experiment was performed in presence of 2.5mM rotenone, an inhibitor of complex I. Subtraction of NADH oxidase activity measured with rotenone to the one measured without represent specific complex I activity.

Carnitine palmitoyl transferase (CPT) LV activity determination was based on a modification of a previously published method (4) and adapted for performing the assays in a 96-well ELISA plate. Briefly, small pieces of LV tissue were homogenized in 40 volumes of a phosphate 0,1M, pH7.2 buffer. The reaction buffer consisted of 0.5 M Tris-HCl pH8, 50 mM EDTA 100 mM L-carnitine and 2.5 mM 5,5'-dithiobis-(2-nitrobenzoic acid) (DTNB). The assay was started with the addition of 180µl of the reaction buffer, 10µl of the homogenate and 10µl of 0.7mM palmitoyl-CoA. The incubation was performed for 5 min at 25°C in a ELISA plate reader fixed at a 412 nM length wave. A measure of the absorbance was made each 15 s during the incubation. The rate of CoA-SH produced by the reaction of the CPT activity and reacting with DTNB and producing the yellow-colored TNB was then calculated.

1. Maltais F, LeBlanc P, Simard C, Jobin J, Berube C, Bruneau J, Carrier L and Belleau R. **Skeletal muscle adaptation to endurance training in patients with chronic obstructive pulmonary disease.** *Am J Respir Crit Care Med* 1996;**154**: 442-447.

2. Maltais F, Simard AA, Simard C, Jobin J, Desgagnés P, LeBlanc P. **Oxidative capacity of the skeletal muscle and lactic acid kinetics during exercise in normal subjects and in patients with COPD.** *Am J Respir Crit Care Med*. 1996;**153**:288-93.

3. Jarreta D, Orus J, Barrientos A, Miro O, Roig E, Heras M, Moraes CT, Cardellach F, Casademont J. **Mitochondrial function in heart muscle from patients with idiopathic dilated cardiomyopathy.** *Cardiovasc Res* 2000;**45**: 860-865.

4. Bieber LL and Markwell MA. **Peroxisomal and microsomal carnitine acetyltransferases.** *Methods Enzymol* 1981;**71** Pt C:351-8.

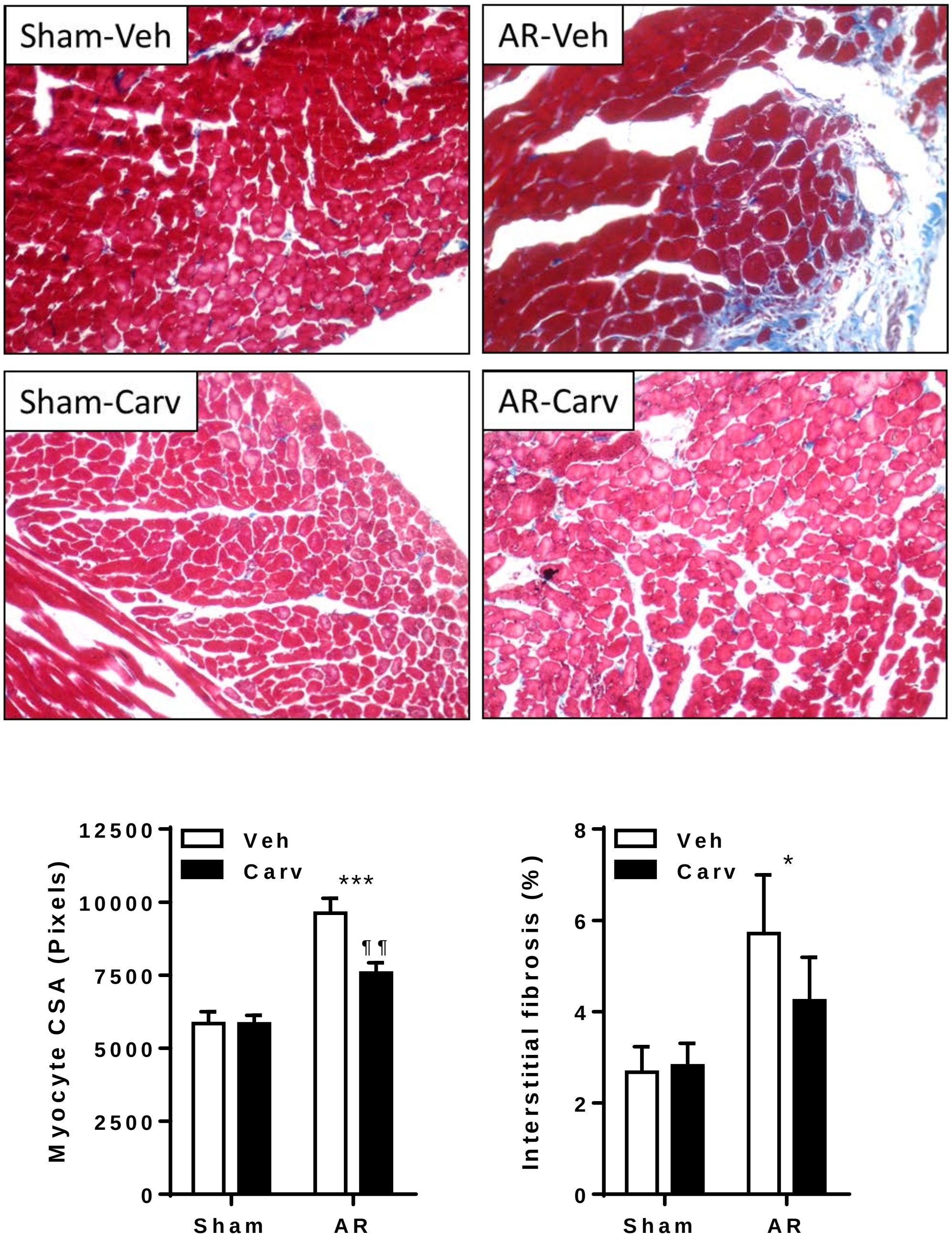

Figure S1. Carvedilol (Carv) reduces cardiac LV myocyte cross-sectional area (CSA) in AR rats. Top panels, typical examples of trichrome-Masson–stained subendocardial LV sections (collagen fibers are blue, cardiomyocytes are red, magnification X200). Bottom left, myocyte cross-sectional area (pixels). Bottom right, quantification of subendocardial fibrosis by blue/red ratio (%) from trichrome-Masson–stained LV sections. Results are reported as mean ± SEM (n=8 to 10). *: p<0.05 and ***: p<0.001 vs. sham animals and ¶¶: p<0.01 vs. vehicle-treated corresponding group.
